# A systematic review on eHealth technology personalization approaches

**DOI:** 10.1016/j.isci.2024.110771

**Published:** 2024-08-19

**Authors:** Iris ten Klooster, Hanneke Kip, Lisette van Gemert-Pijnen, Rik Crutzen, Saskia Kelders

**Affiliations:** 1 Centre for eHealth and Wellbeing Research, Department of Psychology, Health, and Technology, University of Twente, Enschede, The Netherlands; 2 Department of Health Promotion, Care and Public Health Research Institute, Faculty of Health, Medicine and Life Sciences, Maastricht University, Maastricht, the Netherlands; 3 Optentia Research Focus Area, North-West University, Vaal Triangle Campus, Vanderbijlpark, South Africa; 4 Department of Research, Stichting Transfore, Deventer, the Netherlands

**Keywords:** Health sciences, Health technology

## Abstract

Despite the widespread use of personalization of eHealth technologies, there is a lack of comprehensive understanding regarding its application. This systematic review aims to bridge this gap by identifying and clustering different personalization approaches based on the type of variables used for user segmentation and the adaptations to the eHealth technology and examining the role of computational methods in the literature. From the 412 included reports, we identified 13 clusters of personalization approaches, such as behavior + channeling and environment + recommendations. Within these clusters, 10 computational methods were utilized to match segments with technology adaptations, such as classification-based methods and reinforcement learning. Several gaps were identified in the literature, such as the limited exploration of technology-related variables, the limited focus on user interaction reminders, and a frequent reliance on a single type of variable for personalization. Future research should explore leveraging technology-specific features to attain individualistic segmentation approaches.

## Highlights

• Thirteen clusters of eHealth personalization were identified from 412 reviewed studies
• eHealth personalization mainly uses behavioral segmentation like physical activity
• Technology-related variables are missing in eHealth personalization studies
• Predictive modeling and reinforcement learning enable dynamic eHealth adaptations

## Graphical abstract

**Figure:**
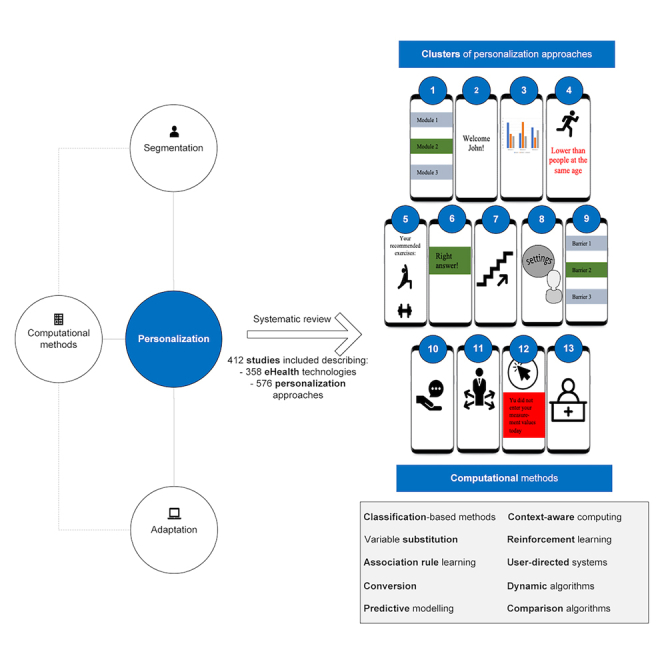

## Introduction

eHealth technologies such as internet-based interventions and mobile apps offer opportunities to make healthcare more effective and efficient and increase health and well-being, but there is room for improvement in terms of their effectiveness.^1^^,^^2^ Personalization offers opportunities to better match technology to individual users, making eHealth technologies more engaging and in turn, making them more effective.^3^^,^^4^^,^^5^ These approaches offer an alternative to a "one-size-fits-all" approach by adapting eHealth technologies to individual users or user groups in the following way: the eHealth technology gathers data about certain characteristics, such as user behavior, demographics, preferences, or interaction with the eHealth technology. These data include data on physical activity from wearables, diseases from electronic health records, or self-reported data through, for example, questionnaires. Computational algorithms are often used to process data on these variables in order to segment users into groups ranging from very small (one person) to large groups (e.g., all females), depending on the number of segmentations. These segmentations are matched with adaptations of features of a technology such as the content, graphical appearance, functionalities, behavior change strategy, or channel,^6^^,^^7^ resulting in a personalized eHealth technology.

Personalization can be applied to eHealth technologies in various ways. To illustrate, an example is the integration of tailored nutrition information messages in the “Happy Me” smartphone application designed for obesity prevention.^8^ To deliver tailored messages, users are segmented into four groups (precontemplation, contemplation, preparation, action) based on the transtheoretical model of change.^8^ According to this model, users in these four different groups need different kinds of support to change their eating habits. To illustrate, if someone is thinking about change but has not committed yet (contemplation stage), messages should help them understand the importance of healthy eating and encourage them to start, whereas a user who is in the action stage benefits more from messages that support their new habits, offering suggestions on how to eat, social support, and reinforcement management. Thus, the computational method for personalization uses theory-driven if-then rules (i.e., conditional logic) based on shared characteristics of a group of users. Another example is CURATE.AI, a system designed to optimize medication dosing for individuals with hypertension and type II diabetes.^9^ With CURATE.AI, the treatment for each patient is personalized in a data-driven manner based on their unique response to medications. The system utilizes specific data from an individual patient, such as their response to drugs and dosages, to determine a personalized treatment, which means that the system adapts to the specific characteristics of each patient, leading to a more personalized approach to treatment. As demonstrated by these examples, the ways in which eHealth technologies can be personalized and tailored are diverse.

There is considerable variation—not only in the ways in which these technologies can be adapted to user variables but also in the types and number of variables used to segment eHealth users. For example, adapting the delivery channel can be matched to segments based on users’ preferences, but this might also be matched to segments of users that vary in age or eHealth literacy. Previous studies have described the diverse ways of personalization^6^^,^^10^ but do not take into account recent technology advances that expand the options for eHealth personalization. Moreover, prior reviews focusing on tailored and personalized eHealth technologies often focus on singular aspect of personalization,^5^ such as feedback provided by the eHealth technology,^3^^,^^11^ or only on a specific target behavior such as weight loss.^12^ This overlooks the broader spectrum of personalization approaches, including variations in user segmentation and technology adaptations. By conducting a comprehensive examination of existing literature on personalized eHealth technologies, our study aims to bridge this gap. Specifically, we seek to identify and categorize diverse personalization approaches based on segmentation variables and technology adaptations. This categorization provides insights into various personalization approaches, thereby contributing to a deeper understanding of their potential impacts on the effectiveness of eHealth technologies. However, it is important to note that although this study lays the groundwork for such exploration, the assessment of effectiveness lies beyond the scope of this paper.

Furthermore, because of the unique opportunities that technology offers over face-to-face interventions, attention is paid to computational methods that are used to translate data on user variables into personalization strategies. The terms “tailoring” and “personalization” are often used interchangeably, and both can be described in terms of segmentation and adaptation.^6^ For this reason, in the current study we use the terms tailoring and personalization together and refer to them as personalization in the remainder of this manuscript.

To reach the aims of the current study, we have formulated the following research questions:

(1) How are users segmented, and what adaptations are incorporated into personalized eHealth technologies?
(2) How can personalization approaches in eHealth be categorized based on user segmentation variables and technology adaptations?
(3) Which computational methods are utilized to match user segmentation variables with technology adaptations?

### Methods

#### Literature search

In this systematic review, an electronic literature search was conducted through the databases Scopus, PubMed, EMBASE, PsycINFO, and IEEE Xplore using a combination of “Personalization,” “Tailoring,” and “eHealth” (see Appendix A for the full search string), with no date restrictions. Since the field of eHealth is multidisciplinary, databases focusing on medical, technical as well as social sciences were included.

The search was updated in ASReview^13^ on 30 February, 2023, because this software became available while undertaking the review. The labeled data from the first full-text screening were used as input to the Naive Bayes classifier to rearrange the records. One author screened the rearranged records using ASReview (I.t.K.). After 50 records were labeled as irrelevant, the screening was stopped, and the records labeled as relevant were imported in Covidence for full-text screening. The full texts were screened by one author (I.t.K.), and a second author (H.K. or S.K.) was consulted in case of doubt.

### Inclusion and exclusion criteria

The inclusion criteria for this review encompassed (1) peer-reviewed journal articles and conference papers describing evaluation studies in which (2) personalized eHealth technologies are described that use technology to change (determinants of) behaviors(s) to improve health, well-being, and healthcare; (3) the eHealth users are segmented in at least two groups, and it is described which variables are used for dividing into user segments; (4) there are adaptations of the technology aligned with the user segments and these adaptations are described; (5) it is described how the segmentations are matched with adaptations and this is computerized; (6) the full text is available in English, Dutch, or German; and (7) the study randomly assigned participants to their condition, and outcomes were related to health or well-being. This inclusion criterion was used to include eHealth technologies that are in (the final stages of) development and to avoid duplication. When studies did not describe which variable(s) were used to segment the users or when the adaptations were not described, the references to which the authors referred to as a more elaborative description of the eHealth technology were screened for a description of the segmentation and/or adaptation used. If there was not a description of segmentation and/or adaptation in those references, or if the authors do not refer to another study, the record was excluded.

After removing duplicates in Covidence, all titles were screened by two authors (I.t.K. and H.K.). If at least one of the authors included a record in the title screening, it was included in the abstract screening. As a next step, the abstracts were screened by the same authors, and differences were discussed until consensus was reached. One author (I.t.K.) then screened all full texts and extracted the data. A second author (H.K. or S.K.) was consulted in case of doubt.

### Data extraction

The data extraction form was based on an adapted version of the Cochrane Data Extraction Form, supplemented with parts of the TIDier checklist^14^ to extract information about the eHealth technology. The extracted data included (1) general information about the study (e.g., author, year of publication), (2) information about the personalized eHealth technology (e.g., type of technology, target group of the eHealth technology), (3) variables used to define user segments, (4) which part(s) of the eHealth technology was adapted and how this was adapted, and (5) the computational method utilized to match user segments with adaptations. The data extraction was carried out by one author (I.t.K.), and a second author was consulted in case of any doubts (H.K. or S.K.). After completing the data extraction, studies that described the same eHealth technology (same name or description) and had at least one overlapping author between studies were merged to prevent overlap within records.

### Data synthesis and statistical analysis

The extracted data were analyzed to identify the segmentations, adaptations, and computational methods used. Moreover, the personalization approaches were categorized based on similarities in the segmentations and adaptations used. Further details about data coding and clustering can be found in the STAR Methods section.

## Results

### Identification of relevant studies

**Figure 1:**
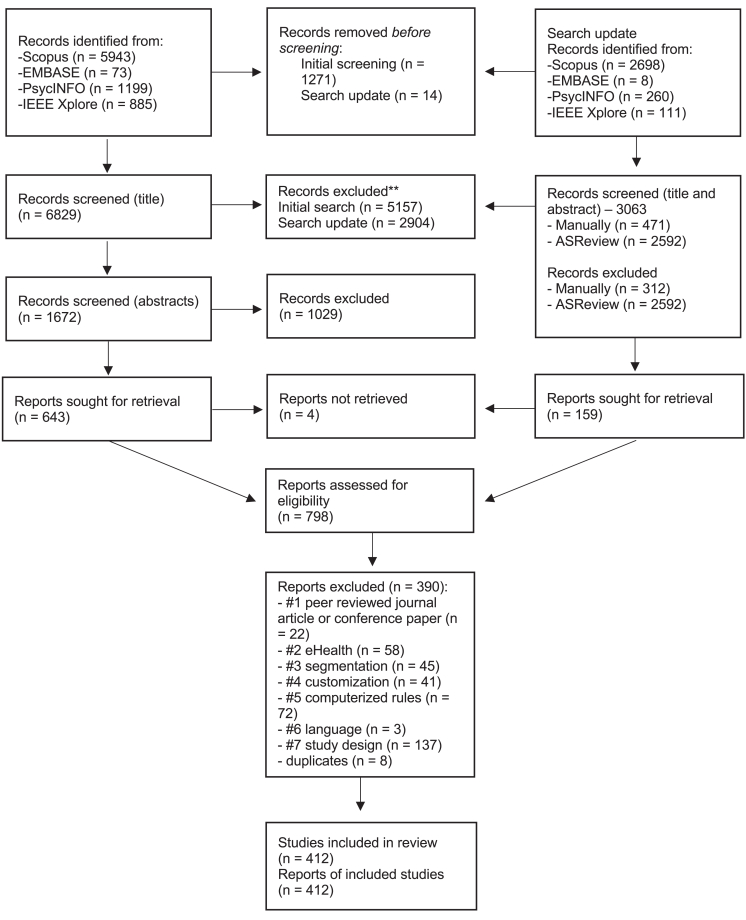
PRISMA flow diagram

Searches of the five databases identified 11,177 references (8,100 in the initial search and 3,077 in the updated search). After screening all records, 802 full-text reports were assessed for eligibility, and 412 reports were included in the systematic review (see Figure 1 for the PRISMA flow diagram^15^).

**Figure 2:**
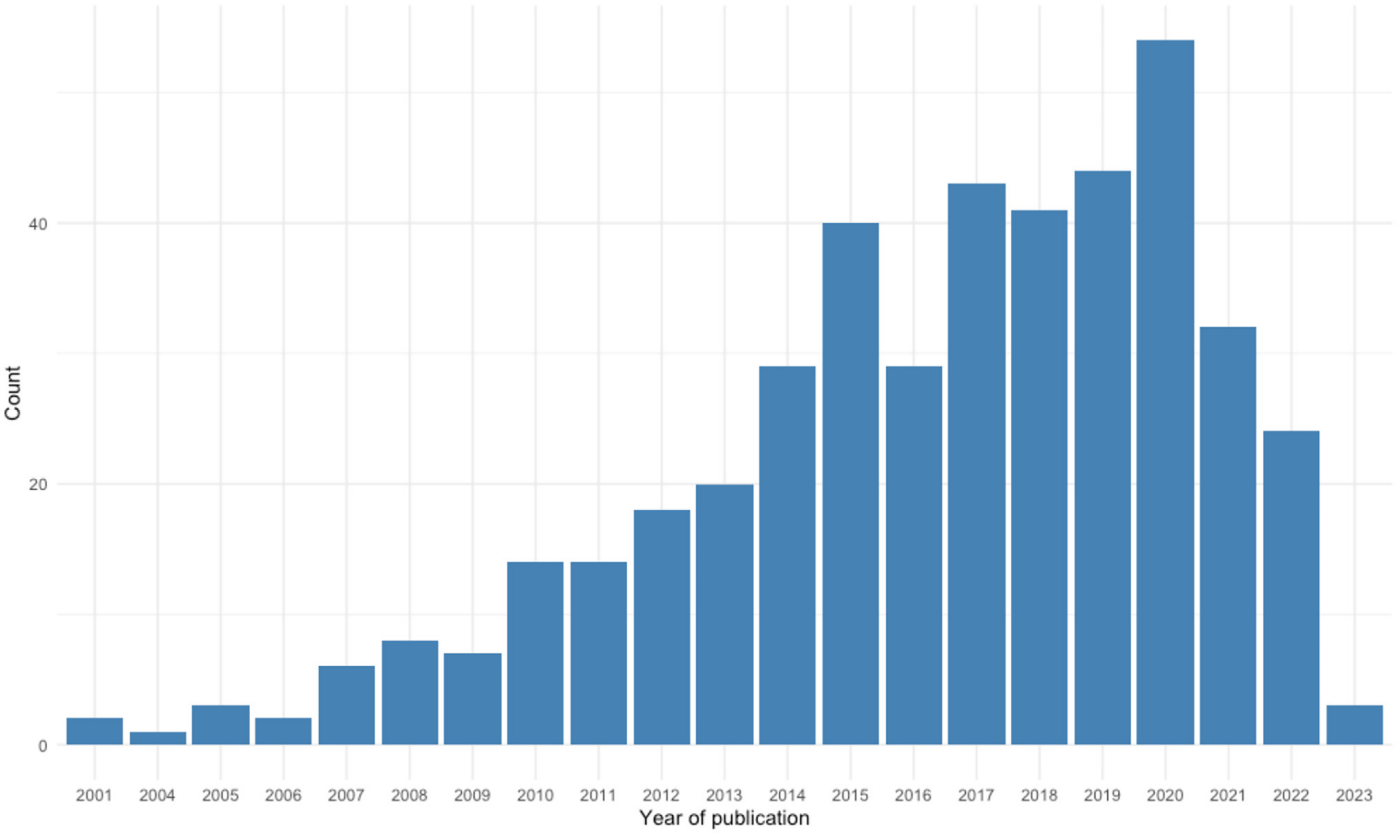
Publication years of included studies

All included studies were published after 2000, with a peak in 2020 (*n* = 54) (see Figure 2).

The included studies described 358 distinct eHealth technologies, with 93 eHealth technologies focusing on substance use^16^^,^^17^^,^^18^^,^^19^^,^^20^^,^^21^^,^^22^^,^^23^^,^^24^^,^^25^^,^^26^^,^^27^^,^^28^^,^^29^^,^^30^^,^^31^^,^^32^^,^^33^^,^^34^^,^^35^^,^^36^^,^^37^^,^^38^^,^^39^^,^^40^^,^^41^^,^^42^^,^^43^^,^^44^^,^^45^^,^^46^^,^^47^^,^^48^^,^^49^^,^^50^^,^^51^^,^^52^^,^^53^^,^^54^^,^^55^^,^^56^^,^^57^^,^^58^^,^^59^^,^^60^^,^^61^^,^^62^^,^^63^^,^^64^^,^^65^^,^^66^^,^^67^^,^^68^^,^^69^^,^^70^^,^^71^^,^^72^^,^^73^^,^^74^^,^^75^^,^^76^^,^^77^^,^^78^^,^^79^^,^^80^^,^^81^^,^^82^^,^^83^^,^^84^^,^^85^^,^^86^^,^^87^^,^^88^^,^^89^^,^^90^^,^^91^^,^^92^^,^^93^^,^^94^^,^^95^^,^^96^^,^^97^^,^^98^^,^^99^^,^^100^^,^^101^^,^^102^^,^^103^^,^^104^^,^^105^^,^^106^^,^^107^^,^^108^^,^^109^^,^^110^^,^^111^^,^^112^^,^^113^^,^^114^^,^^115^^,^^116^^,^^117^^,^^118^^,^^119^^,^^120^^,^^121^^,^^122^^,^^123^^,^^124^^,^^125^^,^^126^^,^^127^^,^^128^^,^^129^^,^^130^^,^^131^^,^^132^^,^^133^^,^^134^^,^^135^; 48 on physical activity^136^^,^^137^^,^^138^^,^^139^^,^^140^^,^^141^^,^^142^^,^^143^^,^^144^^,^^145^^,^^146^^,^^147^^,^^148^^,^^149^^,^^150^^,^^151^^,^^152^^,^^153^^,^^154^^,^^155^^,^^156^^,^^157^^,^^158^^,^^159^^,^^160^^,^^161^^,^^162^^,^^163^^,^^164^^,^^165^^,^^166^^,^^167^^,^^168^^,^^169^^,^^170^^,^^171^^,^^172^^,^^173^^,^^174^^,^^175^^,^^176^^,^^177^^,^^178^^,^^179^^,^^180^^,^^181^^,^^182^^,^^183^^,^^184^^,^^185^^,^^186^^,^^187^^,^^188^^,^^189^^,^^190^^,^^191^^,^^192^^,^^193^^,^^194^^,^^195^^,^^196^^,^^197^^,^^198^^,^^199^^,^^200^^,^^201^^,^^202^; 27 on self-management and self-monitoring^203^^,^^204^^,^^205^^,^^206^^,^^207^^,^^208^^,^^209^^,^^210^^,^^211^^,^^212^^,^^213^^,^^214^^,^^215^^,^^216^^,^^217^^,^^218^^,^^219^^,^^220^^,^^221^^,^^222^^,^^223^^,^^224^^,^^225^^,^^226^^,^^227^^,^^228^^,^^229^^,^^230^^,^^231^^,^^232^^,^^233^^,^^234^^,^^235^^,^^236^^,^^237^^,^^238^^,^^239^^,^^240^; 27 on weight^241^^,^^242^^,^^243^^,^^244^^,^^245^^,^^246^^,^^247^^,^^248^^,^^249^^,^^250^^,^^251^^,^^252^^,^^253^^,^^254^^,^^255^^,^^256^^,^^257^^,^^258^^,^^259^^,^^260^^,^^261^^,^^262^^,^^263^^,^^264^^,^^265^^,^^266^^,^^267^^,^^268^^,^^269^^,^^270^^,^^271^^,^^272^; 20 on dietary behavior^273^^,^^274^^,^^275^^,^^276^^,^^277^^,^^278^^,^^279^^,^^280^^,^^281^^,^^282^^,^^283^^,^^284^^,^^285^^,^^286^^,^^287^^,^^288^^,^^289^^,^^290^^,^^291^^,^^292^^,^^293^^,^^294^; 16 on stress, mental health, and well-being^295^^,^^296^^,^^297^^,^^298^^,^^299^^,^^300^^,^^301^^,^^302^^,^^303^^,^^304^^,^^305^^,^^306^^,^^307^^,^^308^^,^^309^^,^^310^^,^^311^^,^^312^^,^^313^^,^^314^; 15 on depression and anxiety^315^^,^^316^^,^^317^^,^^318^^,^^319^^,^^320^^,^^321^^,^^322^^,^^323^^,^^324^^,^^325^^,^^326^^,^^327^^,^^328^^,^^329^^,^^330^^,^^331^^,^^332^^,^^333^; 13 on adherence^334^^,^^335^^,^^336^^,^^337^^,^^338^^,^^339^^,^^340^^,^^341^^,^^342^^,^^343^^,^^344^^,^^345^^,^^346^^,^^347^^,^^348^^,^^349^^,^^350^^,^^351^^,^^352^; 13 on cardiovascular factors^353^^,^^354^^,^^355^^,^^356^^,^^357^^,^^358^^,^^359^^,^^360^^,^^361^^,^^362^^,^^363^^,^^364^^,^^365^^,^^366^^,^^367^^,^^368^; 9 on testing and vaccination uptake^369^^,^^370^^,^^371^^,^^372^^,^^373^^,^^374^^,^^375^^,^^376^^,^^377^^,^^378^^,^^379^; 8 on safety behaviors^380^^,^^381^^,^^382^^,^^383^^,^^384^^,^^385^^,^^386^^,^^387^^,^^388^; 8 on sleep^389^^,^^390^^,^^391^^,^^392^^,^^393^^,^^394^^,^^395^^,^^396^^,^^397^^,^^398^; 7 on screening^399^^,^^400^^,^^401^^,^^402^^,^^403^^,^^404^^,^^405^^,^^406^^,^^407^; 4 on sedentary behavior^408^^,^^409^^,^^410^^,^^411^^,^^412^; 3 on gambling^413^^,^^414^^,^^415^; 3 on eating disorders^416^^,^^417^^,^^418^^,^^419^; 2 on partner violence^420^^,^^421^^,^^422^^,^^423^^,^^424^^,^^425^; 23 on one type of other behavior^426^^,^^427^^,^^428^^,^^429^^,^^430^^,^^431^^,^^432^^,^^433^^,^^434^^,^^435^^,^^436^^,^^437^^,^^438^^,^^439^^,^^440^^,^^441^^,^^442^^,^^443^^,^^444^^,^^445^^,^^446^^,^^447^^,^^448^^,^^449^^,^^450^^,^^451^^,^^452^^,^^453^^,^^454^^,^^455^^,^^456^^,^^457^^,^^458^^,^^459^^,^^460^^,^^461^; and 20 on multiple health behaviors.^462^^,^^463^^,^^464^^,^^465^^,^^466^^,^^467^^,^^468^^,^^469^^,^^470^^,^^471^^,^^472^^,^^473^^,^^474^^,^^475^^,^^476^^,^^477^^,^^478^^,^^479^^,^^480^^,^^481^^,^^482^^,^^483^^,^^484^^,^^485^ There were 576 personalization approaches described across the 358 eHealth technologies. Descriptions of these personalization approaches can be found in Tables 1 and 2.

**Table 1:** Number of segmentation variables per personalization approach

Number of segmentation variables a	N (%)
1 type of segmentation variable	401 (69.62)
2 types of segmentation variables	131 (22.74)
3 types of segmentation variables	25 (4.34)
4 types of segmentation variables	13 (2.26)
5 types of segmentation variables	6 (1.04)

a Per personalization approach.

**Table 2:** Number of personalization approaches per eHealth technology

Number of personalization approaches a	N (%)
1	204 (56.51)
2	117 (32.41)
3	28 (7.76)
4	9 (2.49)
>4	3 (0.83)

a Per distinct eHealth technology.

### How are users segmented, and what adaptations are incorporated into personalized eHealth technologies?

Among the 576 personalization approaches, most personalization approaches used one type of variable for user segmentation (*n* = 401). In contrast, the number of personalization approaches using four or five types of segmentation variables is 13 (2.26%) and 6 (1.04%), respectively.

**Figure 3:**
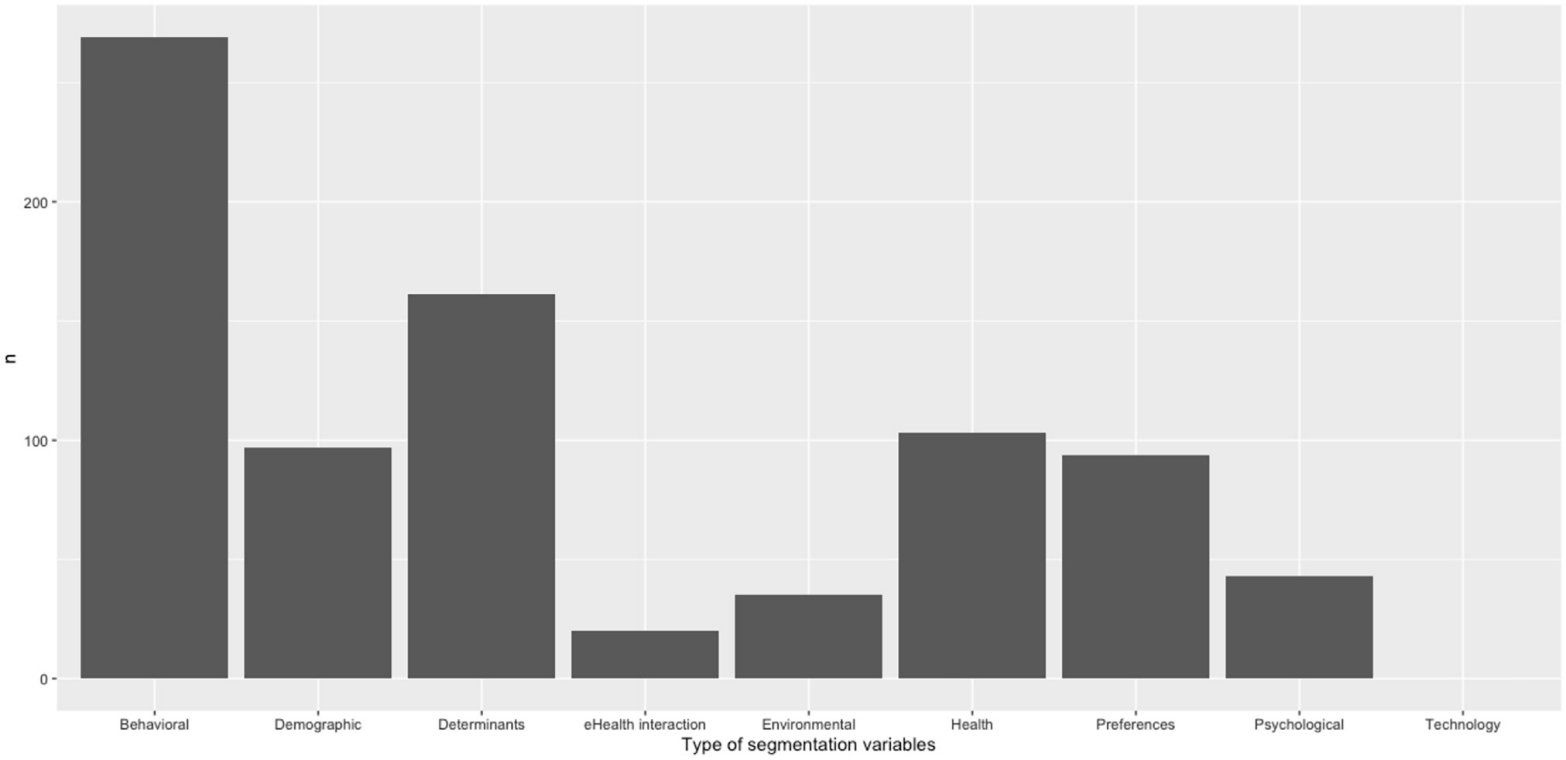
Types of variables used for segmentation

The number of times the different types of variables were used for user segmentation for personalization can be found in Figure 3. User segmentation was mainly based on behavioral variables (*n* = 269) such as vegetable consumption and sun protection behaviors and information about individual determinants (*n* = 161) such as stage of change and perceived behavioral control. Contrastingly, technology-related variables such as digital skills were not used for user segmentation. Moreover, data about the eHealth interaction (*n* = 20) such as whether participants viewed their feedback and environmental information (*n* = 35) such as location and the season were lowest frequently utilized for user segmentation. In Table S22, more examples of segmentation variables can be found per variable type.

The adaptations used in the personalization approaches can be found in Tables 3, 4, 5, 6, and 7. Adaptations to the eHealth technologies were mainly related to the content (*n* = 323), such as providing the user comparative feedback (*n* = 116) or advice (*n* = 91) based on the data that were collected from the user. On the contrary, graphical aspects (*n* = 7) such as including adapted avatars or background pictures and adapting the provision of certain functionalities (*n* = 5) such as including or excluding self-monitoring functionalities were least used in the personalization approaches.

**Table 3:** Types and number of content adaptations and their descriptions

Content adaptations (n)	Description
Comparative feedback (116)	Comparison of current or past data with previous data (ipsative feedback), guidelines, norms (normative feedback), recommendations or individuals who have successfully adopted the target behavior.
Advice (91)	Based on the users' data, an advice is given through the eHealth technology (e.g., how to change the target behavior, advice on which meals to prepare).
Reflective feedback (41)	Feeding back data from the user in which a value is given to the data (e.g., right or wrong answers or perceptions about a certain topic, reinforcement of positive coping strategies).
Interpretative feedback (29)	Feeding back data to the user with an interpretation of the users' data (e.g., money spent on cigarettes based on smoking behavior or risk level for disease(s)).
Feedback (28)	Feeding back data from the user without an interpretation or comparison, such as totals, means, or directly feeding back data from the user (e.g., answers to questions: “You indicated that … ” or mean number of steps per day).
Adapted difficulty level (18)	Content of the eHealth technology provides a difficulty level that is adapted to the data from the user, e.g., step goal or intensity of exercises.

**Table 4:** Types and number of channeling adaptations and their descriptions

Channeling adaptations (n)	Description
Identification (41)	Inclusion of identifying information in the eHealth technology, such as including the name (“Welcome Kate!”), doctor’s name (“Your doctors' name is.”), other static information (“You indicated that you have diabetes”), or testimonials with similar characteristics.
Inclusion or exclusion (39)	Including or excluding information or parts of the eHealth technology based on users' data, not related to whether certain parts are delivered to change the behavior change strategy of the eHealth technology.
Tunneling (27)	Provide advice on which part of the eHealth technology to use or automatically direct the user to parts of the eHealth technology that are relevant to the user based on segmentation variables.
Reminders (19)	Remind users based on users' data (e.g., reminder to visit the eHealth technology, reminder for taking medication).
Delivery timing (18)	Deliver content or parts of the intervention at a specific time point, not related to exceeding a certain threshold (e.g., deliver parts of the intervention at their preferred time).
Frequency (10)	Adapt the frequency that the eHealth technology is delivered to the user.
Alarms (10)	Inform users about exceeding a certain threshold (e.g., increased risk for relapse).
Sequence (5)	Adapt the order in which different parts of the intervention are delivered to the user.
Delivery channel (4)	Adapting the type of channel through which the intervention is delivered based on user’s data (e.g., via text or avatar).

**Table 5:** Types and number of adaptations to the behavior change strategy and their descriptions

Adaptations to the behavior change strategy (n)	Description
Stage matching (32)	Adapting the behavior change strategy based on the process of change of the user, such as messaging tips and tricks around the users' quit date and motivational messages for users who did not set a quit date, or focus on benefits of behavior change in the precontemplation stage.
Target (24)	Adapting the way in which the eHealth technology changes (determinants) of behavior, such as a focus on either increasing knowledge or self-efficacy or providing suggestions for overcoming barriers that the user identified.
Framing (8)	Adapting the eHealth technology by using words, other type of content, or in-depth or concise information in such a way that several aspects of what is described are implicitly highlighted with the assumption that this improves the behavior change strategy.

**Table 6:** Types and number of graphical adaptations and their descriptions

Graphical adaptations (n)	Description
Similarity (7)	Adapting graphical aspects (e.g., avatars, background pictures) of the eHealth technology so that the user identifies with graphical aspects of the eHealth technology.
Structure of data representation (2)	Represent data from the user in such a way that the graphical representations (e.g., tables and figures) are adapted to the data of the eHealth user.

**Table 7:** Types and number of functionality adaptations and their descriptions

Functionality adaptations (n)	Description
Self-monitoring (3)	eHealth technology either provides functionalities for self-monitoring or does not provide this functionality for the user.
Support (1)	eHealth technology either provides functionality that allows for social support or does not provide this functionality for the user.
Text message reminders (1)	eHealth technology either sends text message reminders to the user or does not send these text message reminders.

### How can personalization approaches in eHealth be categorized based on user segmentation variables and technology adaptations?

**Figure 4:**
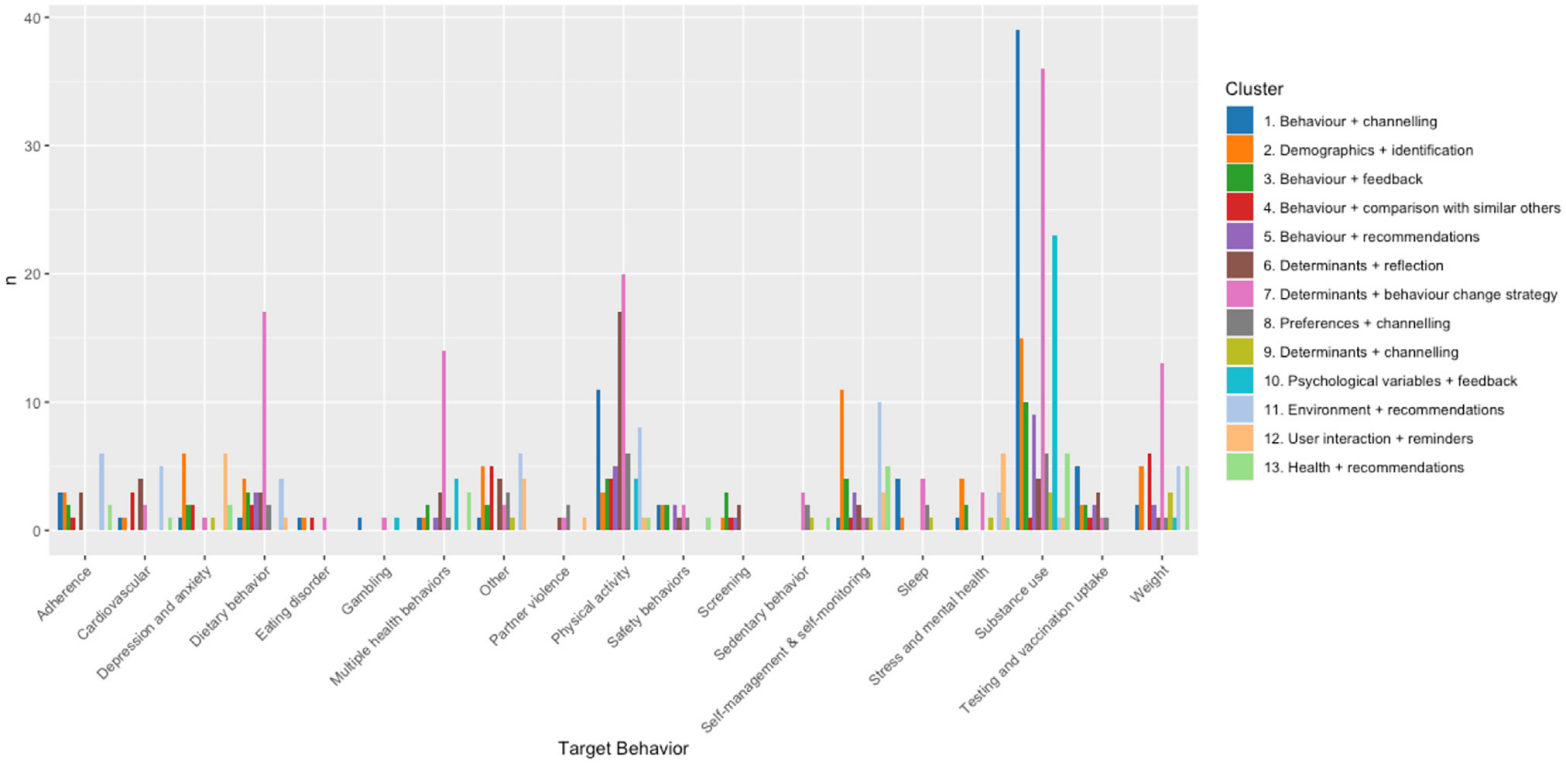
Target behaviors for each cluster

Thirteen clusters were identified through the hierarchical cluster analysis. Cluster names indicate the primary segmentation variable and main adaptation type used for the personalization approaches. Figure 4 provides an overview of the number of personalization strategies per target behavior that fall within each cluster.

#### Cluster 1: Behavior + channeling (n = 28)

Personalization approaches in cluster 1 can be characterized by the use of behavioral segmentation variables, such as sedentary behavior^408^ and exercise and nutrition habits.^323^ These behavior data are used to adapt *what* is delivered through the eHealth technology and *when* this is delivered. An example is a workers' health surveillance module,^295^ in which a tailored set of modules is offered on the basis of behavioral data (such as risky drinking behavior) and psychological data (such as stress and work-related fatigue).

#### Cluster 2: Demographics + identification (n = 38)

Cluster 2 includes eHealth personalization that use demographics about the user, such as names,^384^^,^^462^ gender,^467^ and age.^371^ These data are used to make the technology more recognizable for individual users by, for example, including the name of the user. An example is MyHealthBehaviour,^462^ in which users are addressed by their name in feedback messages.

#### Cluster 3: Behavior + feedback (n = 121)

Almost all personalization and tailoring in this cluster use behavioral variables for segmenting users, such as eating habits.^464^ These behavior data about the user are used to provide feedback (e.g., comparison with food recommendations,^464^ feedback on the personal, financial, and health impact of their smoking^98^) and recommendations (e.g., provide tips on how to change low-scoring goals^241^ and provide light or intensive exercise instructions^297^). An example is MyPlan,^463^ in which users fill out a questionnaire on fruit or vegetables intake or physical activity, which in turn is compared with health norms (comparative feedback). At a later stage this health behavior again is compared to their previous answers to the questionnaire (comparative feedback).

#### Cluster 4: Behavior + comparison with similar others (n = 33)

eHealth personalization in cluster 4 can be characterized by the use of behavioral variables (such as alcohol and drug use^26^) and demographic variables (such as age, gender, and country of origin^35^) for user segmentation. These data are used to provide feedback in which the behavior of the user is compared to behavior of others who have similar demographic characteristics. A personalization from PNC-txt^118^ that falls within this cluster compares the cannabis use of the user with cannabis use of people the same age.

#### Cluster 5: Behavior + recommendations (n = 29)

Personalization in cluster 5 can be characterized by the use of behavioral data (such as frequency of binge eating^416^) for segmentation, combined with one or more other types of variables (such as stress and fatigue^221^ and occupational exposures^436^). Adaptations in this cluster are mainly related to recommendations, such as advice and adapted difficulty levels. An example is the provision of an exercise routine on one of seven exercise levels based on exercise experience (behavioral) and pain (health).^442^

#### Cluster 6: Determinants + reflection (n = 75)

Personalization approaches in cluster 6 use determinants for segmentation, for example readiness to change and self-efficacy^186^ and pain barriers and tendency to catastrophize pain.^205^ eHealth personalization is mainly related to comparative feedback and reflective feedback in which a value is given to the data (e.g., correcting misperceptions about quitting smoking^100^). An example is an HPV-vaccination eHealth technology,^370^ in which mothers indicate their estimated risk of their daughter being infected with HPV. This estimation is used to provide tailored feedback in which an overview is given of the true risk by a virtual assistant to reflect on their estimated risk.

#### Cluster 7: Determinants + behavior change strategy (n = 48)

Cluster 7 includes eHealth personalization that can be characterized by the use of individual determinants for user segmentation. Examples are perceived susceptibility, perceived severity, perceived benefits, perceived barriers to action, cues to action and self-efficacy^381^ (based on the Health Belief Model), and the stage of change of the user^465^ (based on the transtheoretical model). These segmentations are translated into adaptations to the behavior change strategy of the eHealth technology. An example is stage matching, which is operationalized in Step Advice as having a different approach for each of the five stages of change. As such, general information was provided to users in the precontemplation stage, whereas information for users in the action stage focused on preventing relapse.^143^ Additionally, eHealth personalization in this cluster regularly target (such as offering messages positively influencing attitudes, subjective norms, perceived behavioral control, or intention regarding physical activity^152^) and use framing (such as providing either promotion messages that are framed to promote the benefits of performing regular physical activity or prevention messages in which the health problems that can be avoided by performing regular physical activity are highlighted^182^) to adapt eHealth technologies.

#### Cluster 8: Preferences + channeling (n = 65)

Personalization in cluster 8 can be characterized by the use of preferences for user segmentation, such as preference for receiving messages from the avatar or text only,^383^ preferences related to the timing of messages,^327^ and preferences concerning which modules to follow.^321^ These data are used to adapt the channeling of the eHealth technology, such as either providing eHealth modules with a focus on problem solving or practice exposition to fear inducing situations^321^ or delivering text messages at the user’s preferred time.^327^ An example is the inclusion of avatars in Alerta Alcohol,^67^ which is adapted to the users’ choice for an avatar and their preferred name.

#### Cluster 9: Determinants + channeling (n = 28)

Personalization in cluster 9 mainly use determinants (such as perceived competence^181^ and personal attitudes about vaccination^379^), sometimes combined with behavioral variables (such as dieting status^276^) for segmentation. Adaptations are mainly related to channeling of the eHealth technology. For example, providing tips on the most challenging behaviors^245^ (inclusion or exclusion) or providing support when craving levels for drinking is high.^42^ Another example of tailoring within this cluster is used in Vaccines and Your Baby,^379^ in which users’ values about vaccination are used to determine how tiles on the website are arranged (tunneling). More specifically, in “Just for You,” top three piles contain the most relevant content on the basis of the user’s values.

#### Cluster 10: Psychological variables + feedback (n = 23)

Cluster 10 can be characterized by the use of psychological variables for segmentation, such as mood and daily stress,^304^ monitoring or blunting coping style,^289^ and symptoms of social anxiety.^318^ Adaptations are mainly related to feedback and channeling of the eHealth technology. An example of a personalization within this cluster is Kelaa,^303^ in which users’ stress, well-being, and resilience (psychological variables) are used for segmentation. Users receive feedback on these psychological variables and advice on what they can do about this. Another example is CBTpsych.com,^318^ in which the user ranks thoughts and behaviors on how much they are relevant for their social anxiety that is used to decide on the course of treatment (sequence).

#### Cluster 11: Environment + recommendations (n = 28)

Cluster 11 includes personalization that use environmental variables (such as the season,^466^ the weather,^171^ and cannabis use in the users’ peer network^118^), regularly combined with preferences (such as the users’ tastes in food,^466^ exercise preferences, ^451^ and preference for advice with or without medication^210^). These variables are mainly translated in advice, such as providing recommended HIV testing facilities near the users’ location^377^ and providing exercise recommendations in line with the preferences of the user.^451^ Another example is the ANODE program,^466^ which generates menus (advice) on the basis of the users’ preferences and the season (environmental).

#### Cluster 12: User interaction + reminders (n = 12)

Personalization in cluster 12 can be characterized by using only user interaction information for user segmentation. Examples are whether the user had logged any challenges^300^ and whether the electronic diary within the eHealth technology was used.^264^ Adaptations mainly include reminders and very infrequently tunneling. Examples are reminders to use the eHealth technology and to enter measurement values^242^ and highlighting website components the user did not navigate to (tunneling).^85^

#### Cluster 13: Health + recommendations (n = 48)

Personalization in cluster 13 can be characterized by the use of health variables for user segmentation, such as the user’s fitness level,^156^ ALDH2 genotype,^62^ and symptoms and medical history.^210^ These health data are mainly used to provide recommendations, such as providing dietary advice^275^ and providing adapted entry levels regarding the intensity of the exercise regimen.^275^ Other examples are sending medication reminders at the user’s specified dosing time^340^ and safety alerts when glucose or blood pressure levels or weight exceed a certain threshold.^231^

### Which computational methods are utilized to match user segmentation variables with technology adaptations?

We identified several computational methods in the eHealth personalization approaches based on inductive coding. Various computational methods were utilized to match segments to technology adaptations, namely classification-based methods (*n* = 216), comparison algorithms (*n* = 128), variable substitution (*n* = 64), user-directed systems (*n* = 47), dynamic algorithms (*n* = 54), conversion (*n* = 26), context-aware computing (*n* = 23), reinforcement learning (*n* = 4), predictive models (*n* = 13), and association-rule learning (*n* = 1). The descriptions of these computational methods and examples can be found in Table 8.

**Table 8:** Computational methods to match segmentations to adaptations

Computational method (n)	Description	Examples
Classification-based methods (213)	Computational methods utilized to allocate or assign users to specific groups or interventions based on predefined criteria, such as group assignment and decision rules.	Assigning smokers to a group that receives one smoking cessation message per week^227^ (cluster 1); algorithm assigns specific messages to individuals based on their age group (e.g., messages for individuals aged 12–17 y focus on topics such as college applications^308^) (cluster 2); content differed between stages (e.g., precontemplators mainly received general information about the 10,000 steps concept)^143^ (cluster 7).
Comparison algorithms (129)	Computational methods employed to compare individual user’s current behaviors with guidelines or norms or compare segmentation variables with each other (e.g., Analytic Hierarchy Process).	Participants who reported consuming fewer than five green foods received more messages encouraging fruit and vegetable consumption in proportion to their reported behavior^266^ (cluster 1).
Variable substitution (65)	Replacing placeholders based on segmentation variables in a textual format or with visual representations.	Messages are customized by replacing variables such as names and pronouns with gender-matched descriptions of role models, such as Bill for men and Rachel for women.^467^ (cluster 2); dashboard showing key metrics over time (active minutes, miles, steps, stairs, and heart rate zone)^184^ (cluster 5).
Dynamic algorithms (54)	Computational approach that adapts and responds to changes in segmentation variables.	A booster was employed for participants who return to negative behavior after exhibiting positive behavior^203^ (cluster 1); reminders to use the website and after not logging in for 7 days (cluster 12).
User-directed systems (47)	Computational method that allows the user to directly control the system’s behavior, functions, and features.	During the sign-up process, users are prompted to specify their gender, first name, and their preferred conversation style in Dutch. This style choice includes distinctions between formal and informal conversation forms, which are adapted based on specific display rules^276^ (cluster 8).
Conversion (27)	Applying a conversion factor or formula for an estimation based on the given segmentation variables.	The reported alcohol consumption is converted to the caloric value, and the maximum reported alcohol intake is converted to the blood alcohol concentration along with potential consequences^54^ (cluster 3).
Context-aware computing (23)	Using contextual information, such as location and time to personalize the intervention delivery to the segmentation variables.	Two additional messages were sent at the participants’ heaviest typical drinking times^42^ (cluster 1).
Predictive modeling (13)	Computational method that is used to predict future outcomes based on segmentation variables.	During the initial two weeks, EMA surveys were gathered to personalize the lapse prediction algorithm. Risk alerts were then activated at the onset of the third week. A decision tree algorithm was employed to predict the likelihood of a lapse report in the subsequent EMA survey. The algorithm, based on both group and individual data, classified responses into four risk categories: no risk, low risk, medium risk, and high risk, aiding in timely intervention^267^ (cluster 9).
Reinforcement learning (4)	Adapting the matching of segmentations and adaptations based on the collected segmentation variables to continuously refine the personalization approach.	After clustering user behaviors, MyBehavior uses an exploit-explore strategy to automatically generate suggestions based on users’ past physical activities and food intake^479^ (cluster 3).
Association rule learning (1)	Computational method that uncovers the relationships between segmentation variables to identify patterns where one set of values of segmentation variables tends to appear together.	The FutureMe app employs market-basket analysis to analyze users' recent food purchases and identify food categories (baskets) with the highest potential for improvement in nutritional quality. The app evaluates the negative contributions of these food categories to users' dietary assessments. It then suggests healthier alternatives within these problematic categories, aiming to encourage users to make healthier choices without significantly altering their dietary habits^483^ (cluster 3).

**Figure 5:**
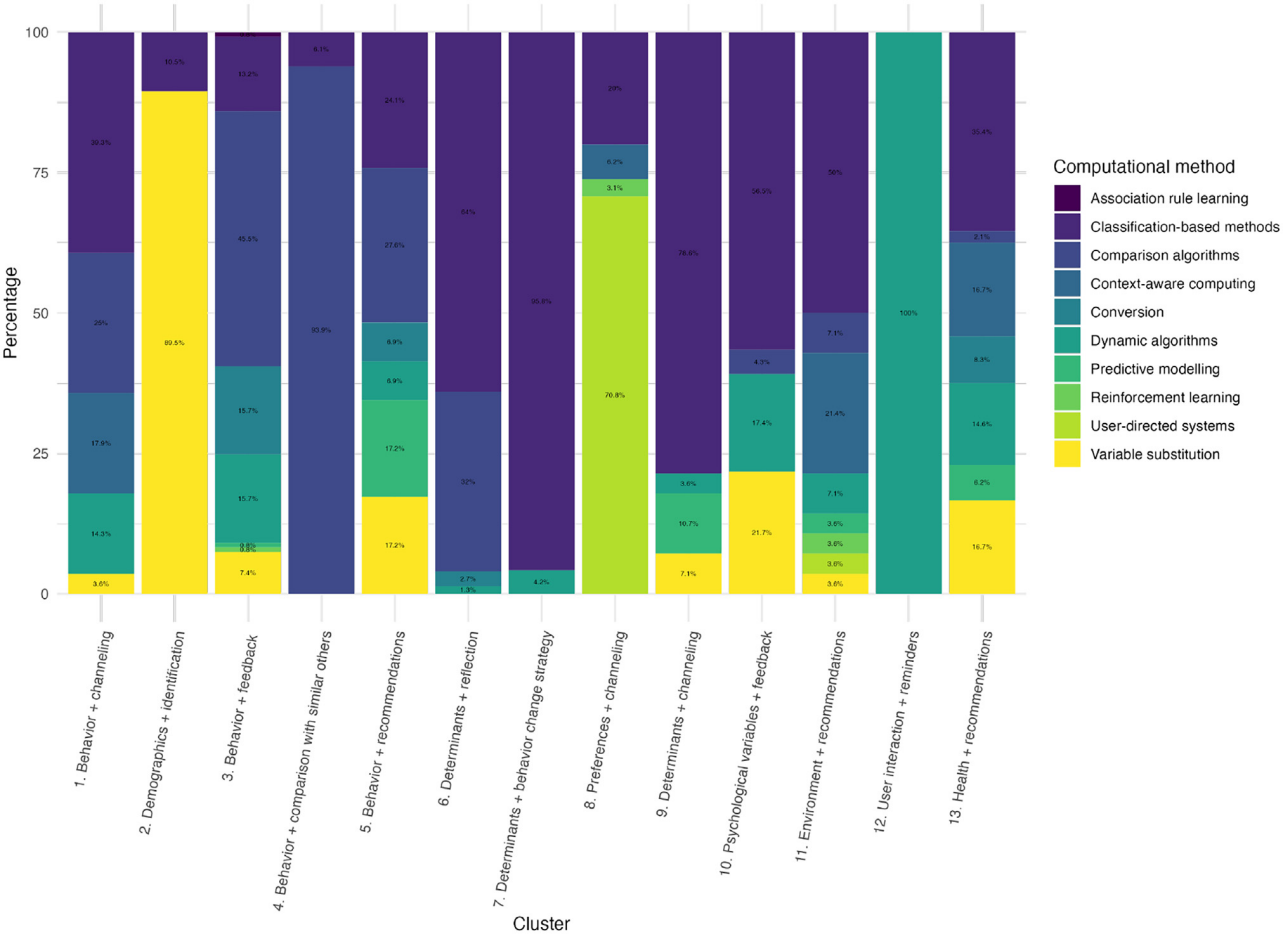
Overview of computational methods by cluster

To gain more insight into the computational methods used per distinct cluster, an overview of the computational methods per cluster can be found in Figure 5 below. We identified six clusters in which a highly prevalent computational method was employed. Cluster 2 (demographics + identification) predominantly utilized variable substitution techniques (89.47%). In cluster 4 (behavior + comparison with similar others), comparison algorithms were predominantly employed (93.94%). For cluster 7 (determinants + behavior change strategy), classification-based methods were the primary computational approach (95.83%). In cluster 8 (preferences + channeling), user-directed systems emerged as the dominant method (70.77%). Similarly, in cluster 9 (determinants + channeling), classification-based methods were predominantly utilized (78.57%). Lastly, cluster 12 (user interaction + reminders) relied on dynamic algorithms (100%).

Clusters 1, 3, 5, 6, 10, 11, and 13 employ a variety of computational methods for personalization. Classification-based methods appear to be widely adopted in these clusters, but also other methods are utilized, such as the comparison algorithms in cluster 1 (*n* = 7), dynamic algorithms in cluster 5 (*n* = 2), conversion in cluster 6 (*n* = 2), variable substitution in cluster 10 (*n* = 5), and context-aware computing in cluster 13 (*n* = 8). Clusters 3 and 11 also utilized more advanced computational methods, such as association-rule learning, reinforcement learning, and predictive modeling.

## Discussion

This study aimed to identify and categorize diverse personalization approaches offering comprehensive insights into the strategies employed for eHealth personalization. With the 412 studies that were included in the current review, we were able to identify 13 clusters of personalization approaches that show similarities in either or both the type of segmentation variables and how the eHealth technology was adapted to these user segments. Overall, we found that most personalized eHealth technologies used behavioral segmentation variables such as alcohol consumption and physical activity, which is in line with a previous meta-analysis on eHealth tailoring.^486^ Similar to previous descriptions of how personalization can be applied in the eHealth design,^6^^,^^487^ we found that eHealth technologies are mainly adapted by providing feedback to the user. In contrast, other clusters that we identified in the current systematic review are, to our knowledge, not so evident in eHealth literature, such as user interaction + reminders, determinants + channeling, and psychological variables + feedback.

We identified several gaps in the literature. First, previous studies have found that eHealth usage is often limited, which is regularly attributed to digital skills.^488^ Yet, none of the included studies used technology-related variables for user segmentation. Technology-related variables encompass a user’s skills and experience with different forms of technology, such as digital literacy and experience with VR technologies.^7^ This absence of technology-related variables for user segmentation is notable, given the potential of these variables to increase adherence and engagement to eHealth technologies.^489^^,^^490^ Moreover, previous studies on adherence to eHealth technologies have found that often only young, educated women are adherent to eHealth technologies,^491^ which might be explained by higher eHealth literacy levels.^492^ Given the promising prospects that personalization holds for increasing both engagement and effectiveness,^493^ it is important to explore how personalizing to these technology-related variables can improve eHealth technologies. Technology-related variables offer opportunities to focus not only on adapting the content of an eHealth technology but also on other aspects such as providing a simple design or incorporating text-to-speech engines for low eHealth literacy users.^494^ In essence, addressing these technology-related variables in personalization strategies could bridge the gap between current usage patterns and the potential for increased engagement and effectiveness across a broader population of users.

Second, user interaction reminders were the least used in the included literature. A possible explanation for this is that the first versions of tailored (non-digital) health communication used printed materials and did not collect data on whether, for example, a user had read certain information or not (e.g.,^495^). In addition, when user interaction segmentation was used in the included studies, only dynamic algorithms were used, such as whether or not a user visited a particular page or not. A more personalized approach toward user interaction can be to segment users according to how their usage changes over time or how they respond to certain behavior change strategies.^496^ More advanced computational methods can be used that better exploit the opportunities for capturing the users’ eHealth interaction in real-time through log data.^497^ For example, reinforcement learning can be utilized to analyze user interaction patterns to determine the most effective types and frequencies of reminders. By continuously observing how users respond to different types of reminders, reinforcement learning algorithms can learn which strategies lead to increased user engagement and adherence to the eHealth technology and adapt the eHealth technology to the individual user accordingly.

Third, the largest part of the included studies only used one type of variable in their eHealth technology for user segmentation. The implementation of personalization based on a single variable fails to capture the complexity inherent in individual behaviors, characteristics, and the process of behavioral change. It is important to recognize that the type and number of variables depend on individual differences,^7^ so there is no “one-size-fits-all” approach or standard set of variables that can be used within each technology. Yet, adopting a multi-faceted approach to user segmentation holds promise in enhancing the efficacy of personalized eHealth interventions. To illustrate, health-related segmentation variables allow for adaptations like personalized recommendations. Integrating these variables with preferences, such as whether the user prefers recommendations with or without medications, might enhance these adaptations. Another approach involves the use of existing behavioral patterns combined with user preferences to adapt suggestions given to the user for increasing physical activity and improving dietary behaviors.^479^

A last gap in literature is related to the computational methods used in the different personalization clusters. We found that the current computational approaches mainly use classification-based methods, which are primarily suited for rather stable characteristics of users or eHealth technologies designed for short-term use. However, there are clusters with more variability in the computational methods employed, such as cluster 3 (behavior + feedback) and cluster 11 (environment + recommendations). This variability likely arises from the dynamic nature of the variables involved, like behavior and environment, which change over time, and the adaptive elements, like recommendations and feedback. For clusters displaying similar patterns, it could be beneficial to evaluate other computational methods. For instance, cluster 7 (determinants + behavior strategy) includes determinants that may change over time, suggesting that alternative computational approaches may offer added value. Thus, although classification-based computational methods might be more suitable in some contexts, it may not be sufficient for more dynamic characteristics or for eHealth technologies designed for long-term use. The use of dynamic algorithms enhances these approaches by collecting segmentation variables over time and adapting to changes in these variables. Yet, these dynamic computational methods do rely on general rules that have to be decided in advance by the designer of the technology. Therefore, these computational methods seem mainly appropriate in contexts in which the matching between segmentations and adaptations remains stable and when the designer of the technology can anticipate what adaptations should be matched with which user segmentations in a meaningful way. More advanced machine learning methods offer opportunities to further refine how segmentations are matched with adaptations in real time. For instance, reinforcement learning techniques allow for adaptations based on segmentation variables collected through the eHealth technology. To illustrate, users can provide feedback on the messages they receive, which in turn is used to “learn” what adaptations are suitable for the individual user, and thus improve the matching of segmentations with adaptation strategies. Moreover, predictive models can be trained on data specific to each individual user (such as finding predictors for certain events that may only account for an individual user). These models learn patterns and behaviors unique to each user, allowing for highly personalized recommendations, predictions, or interventions that take into account individual variation.

### Limitations of the study

A limitation of this systematic review is that the included studies sometimes described the way in which users were segmented differently, which may have affected the clustering results. For example, some studies describe the segmentation variable as “risk of cardiovascular disease” (health variable), whereas another study describes the same in more detail (what constitutes this risk of cardiovascular disease), such as “physical activity,” “smoking” (behavioral), “age” (demographic), and “history of cardiovascular disease” (health). It is essential that in future research, this is reported more explicitly.^498^ The data that were collected from users should be described, followed by a description of the computational method used, in this example, to calculate their risk of cardiovascular disease.

Furthermore, due to the interdisciplinary nature of eHealth research, we chose to include databases that collectively cover a wide range of disciplines, including medicine, biomedical sciences, psychology, engineering, technology, and social sciences, while refraining from multiple databases within a single discipline. Although this approach mitigates potential bias toward a particular field, it is important to acknowledge the risk of omitting valuable records. Furthermore, our inclusion criteria focused specifically on evaluation studies with randomization, potentially overlooking more exploratory studies. For instance, it is likely that more advanced computational methods, which may still be in the exploratory phase, were not fully represented in our analysis.

Lastly, the coding process embraced a collaborative approach, with segmentation and adaptation data partly double-coded (5%) and discrepancies resolved through consensus discussions. This method not only provided an understanding of the strategies used for personalization but also allowed for the incorporation of both existing frameworks and emergent themes, enriching the qualitative analysis. Although this approach facilitated comprehensive insights, it is important to acknowledge that the absence of formal inter-rater reliability assessment introduces some uncertainty regarding coding consistency.

### Future research

A focus on several key areas is needed in future research efforts to advance the field of eHealth personalization. Firstly, we found that personalization approaches are diverse, and their added value can be explained in various ways. To illustrate, eHealth technologies can be personalized based on a single type of variable (e.g., eHealth interaction or preferences), but this can also be done based on multiple different types of variables (e.g., eHealth interaction combined with user preferences). These approaches might differ in the extent to which they increase the effectiveness of eHealth technologies (e.g., using more variables might work better). As such, we argue that future research should explore what effective components of personalization strategies are by employing advanced research methods (e.g., dismantling designs or factorial designs) and moving away from randomized controlled trial (RCT) studies that only take into account whether or not an eHealth technology was personalized to gain more insight into why and for whom certain personalization strategies work. As a preliminary step, our clusters of personalization approaches highlight the divergent approaches of personalization across various applications. This insight can inform future studies aimed at investigating how personalization enhances the effectiveness of eHealth technologies, such as its impact on adherence, engagement, perceived relevance, and other relevant possible working mechanisms.^490^^,^^499^^,^^500^ To illustrate, we believe that for instance, demographics + identification (cluster 2) shows different working mechanisms than cluster 1 in which the user is actually tunneled toward components of the eHealth technology that are presumed to be more relevant to the user based on behavioral segmentation variables.

Second, future research should focus on further exploring how advanced computational methods can be utilized for eHealth personalization. This can be done by examining approaches in other domains outside of eHealth as well as by considering lessons learned from exploratory studies within the field of eHealth itself, which were not included in the current review. It is also important to focus on the underlying theories of these advanced computational methods to explain their added value. By understanding the theoretical foundations, researchers can better articulate why these methods enhance personalization.

### Conclusion

In conclusion, the broad range of clusters of personalization approaches we identified illustrates the multifaceted nature of eHealth personalization. The clusters of personalization approaches can be used as a resource for informing the design process. By comprehending the diverse applications of personalization, designers can integrate this knowledge into the development of eHealth technologies based on specific contextual needs. However, the finding that several possibilities of eHealth personalization have not yet been fully explored, such as the use of technology-related variables for user segmentation and the use of advanced computational methods to match user segmentations with adaptations, illustrates the importance of further research.

## Resource availability

### Lead contact

Further information and requests for resources should be directed to and will be fulfilled by the lead contact, Iris ten Klooster (i.tenklooster@utwente.nl).

### Materials availability

This study did not generate new unique reagents.

### Data and code availability

• Data: The datasets generated during this study are available at OSF (https://doi.org/10.17605/OSF.IO/5W4AR)
• Code: The code used for data analysis is available at OSF (https://doi.org/10.17605/OSF.IO/5W4AR)
• Any additional information required to reanalyze the data reported in this paper is available from the lead upon request.

## Acknowledgments

This work was supported by the Netherlands Organization for Scientific Research (NWO) and partners Podotherapy Center Wender, Reggeborgh, and DIAVASC and Hospital Group Twente (ZGT) (grant number 628.011.024).

## Author contributions

L.G., R.C., H.K., and I.t.K. conceptualized this study. R.C., H.K., and I.t.K. designed the methodology. H.K. and I.t.K. screened the records. S.K. and I.t.K. did the statistical analyses. I.t.K. supervised the screening and data analysis. I.t.K. wrote the draft manuscript with input from H.K. and S.K. All authors contributed to the final manuscript. All authors have read and agreed to the final version of the manuscript. All authors have full access to all data in the study and have a final responsibility for the decision to submit for publication. I.t.K. and S.K. have verified the data.

## Declaration of interests

The authors declare no competing interests.

## STAR★Methods

### Key resources table

**Table:** 

REAGENT or RESOURCE	SOURCE	IDENTIFIER
**Software and algorithms**		

R	CRAN	RRID:SCR_001905
RStudio	RStudio	RRID:SCR_000432
ASReview	ASReview	N/A
dplyr	CRAN	RRID:SCR_016708
tidyr	CRAN	RRID:SCR_017102
Cluster package	CRAN	RRID:SCR_013505
Covidence	Covidence	RRID:SCR_016484
Data	OSF	https://doi.org/10.17605/OSF.IO/5W4AR
Code	OSF	https://doi.org/10.17605/OSF.IO/5W4AR

### Method details

The intended methods were documented on PROSPERO (CRD42021231093) on March 19, 2021 prior to the literature searches, data extraction and data analysis. In this systematic review, an electronic literature search was conducted through the databases Scopus, PubMed, EMBASE, PsycINFO, and IEEE Xplore using a combination of ‘Personalization’, ‘Tailoring’ and ‘eHealth’ (see Appendix A for the full search string), with no date restrictions. Since the field of eHealth is multidisciplinary, databases focusing on medical, technical as well as social sciences were included. This review was reported according to the PRISMA guidelines.^15^

The search was updated in ASReview ^13^ on 30 February, 2023, because this software became available while undertaking the review. The labeled data from the first full text screening was used as input to the Naive Bayes classifier to rearrange the records. One author screened the rearranged records using ASReview (I.t.K.). After 50 records were labeled as irrelevant, the screening was stopped, and the records labeled as relevant were imported in Covidence for full-text screening. The full texts were screened by one author (I.t.K.), and a second author (H.K. or S.K.) was consulted in case of doubt.

The inclusion criteria for this review encompassed (1) peer-reviewed journal articles and conference papers describing evaluation studies in which (2) personalized eHealth technologies are described that use technology to change (determinants of) behaviors(s) to improve health, wellbeing and healthcare, (3) the eHealth users are segmented in at least two groups and it is described which variables are used for dividing into user segments, (4) there are adaptations of the technology aligned with the user segments and these adaptations are described, (5) it is described how the segmentations are matched with adaptations and this is computerized, (6) the full-text is available in English, Dutch or German, (7) the study randomly assigned participants to their condition and outcomes were related to health or wellbeing. This inclusion criterion was used to include eHealth technologies that are in (the final stages of) development and to avoid duplication. When studies did not describe which variable(s) were used to segment the users or when the adaptations were not described, the references to which the authors referred to as a more elaborative description of the eHealth technology were screened for a description of the segmentation and/or adaptation used. If there was not a description of segmentation and/or adaptation in those references, or if the authors do not refer to another study, the record was excluded.

After removing duplicates in Covidence, all titles were screened by two authors (I.t.K. and H.K.). If at least one of the authors included a record in the title screening, it was included in the abstract screening. As a next step, the abstracts were screened by the same authors, and differences were discussed until consensus was reached. One author (I.t.K.) then screened all full texts and extracted the data. A second author (H.K. or S.K.) was consulted in case of doubt.

The data extraction form was based on an adapted version of the Cochrane Data Extraction Form, supplemented with parts of the TIDier checklist ^14^ to extract information about the eHealth technology. The extracted data included (1) general information about the study (e.g., author, year of publication), (2) information about the personalized eHealth technology (e.g., type of technology, target group of the eHealth technology), (3) variables used to define user segments, (4) which part(s) of the eHealth technology was adapted and how this was adapted, and (5) the computational method utilized to match user segments with adaptations. The data extraction was carried out by one author (I.t.K.), and a second author was consulted in case of any doubts (H.K. or S.K.). After completing the data extraction, studies that described the same eHealth technology (same name or description) and had at least one overlapping author between studies were merged to prevent overlap within records.

### Quantification and statistical analysis

The extracted data were split into distinct personalization or tailoring strategies (e.g., an eHealth technology with two personalizations was split into two rows). The variables that were used to define user segments were deductively coded into the categories ‘behavioral’, ‘determinants’, ‘health’, ‘demographic’, ‘preferences’, ‘psychological’, ‘environmental’, ‘eHealth interaction’ and ‘technology’ based our framework developed in a previous study.^7^ Adaptations were coded using a combination of deductive coding into the categories ‘content’, ‘channeling’, ‘behavior change strategy’, ‘graphical’ and ‘functionalities’ (using the same framework), and inductive coding to define subcategories of these categories. These coded combinations of segmentation variables and adaptations were hierarchically clustered in RStudio using the Cluster package.^501^ Gower distances were used since data was both binary (whether a variable was used for segmentation) and categorical (what type of adaptation was used). The number of clusters was determined using incremental clustering, meaning that, starting from two clusters, extra clusters were added until an extra cluster did not add extra information.

## Appendix A. Search string

(personali∗ OR tailor∗) AND (((electronic OR tele∗ OR remote OR online OR web-based OR internet OR technology) W/3 (intervention OR therapy OR "behavio∗r change" OR treat∗)) OR ehealth OR e-health OR m-health OR mhealth OR “mobile health” OR “e-mental health”).

## Supplemental information

Table S1. PRISMA abstract checklist, related to STAR methods ‘Method Details’

Table S2. PRISMA checklist, related to STAR methods ‘Method Details’

Table S3. Excluded full texts, related to figure 1

Table S4. eHealth technologies focusing on testing and vaccination uptake, related to Figure 4

Table S5. eHealth technologies focusing on gambling, related to Figure 4

Table S6. eHealth technologies focusing on safety behaviors, related to Figure 4

Table S7. eHealth technologies focusing on eating disorders, related to Figure 4

Table S8. eHealth technologies focusing on stress, mental health, and well-being, related to Figure 4

Table S9. eHealth technologies focusing on multiple health behaviors, related to Figure 4

Table S10. eHealth technologies focusing on sedentary behavior, related to Figure 4

Table S11. eHealth technologies focusing on depression and anxiety, related to Figure 4

Table S12. eHealth technologies focusing on physical activity, related to Figure 4

Table S13. eHealth technologies focusing on substance use, related to Figure 4

Table S14. eHealth technologies focusing on weight, related to Figure 4

Table S15. eHealth technologies focusing on dietary behavior, related to Figure 4

Table S16. eHealth technologies focusing on self-management and self-monitoring, related to Figure 4

Table S17. eHealth technologies focusing on cardiovascular factors, related to Figure 4

Table S18. eHealth technologies focusing on adherence, related to Figure 4

Table S19. eHealth technologies focusing on sleep, related to Figure 4

Table S20. eHealth technologies focusing on screening, related to Figure 4

Table S21. eHealth technologies focusing on partner violence, related to Figure 4

Table S22. eHealth technologies focusing on other, related to Figure 4

Table S23. Examples of the different types of segmentation variables, related to Figure 3
